# Bioinspired Green Synthesis of Chitosan and Zinc Oxide Nanoparticles with Strong Antibacterial Activity against Rice Pathogen *Xanthomonas oryzae* pv. *oryzae*

**DOI:** 10.3390/molecules25204795

**Published:** 2020-10-19

**Authors:** Yasmine Abdallah, Mengju Liu, Solabomi Olaitan Ogunyemi, Temoor Ahmed, Hatem Fouad, Amro Abdelazez, Chenqi Yan, Yong Yang, Jianping Chen, Bin Li

**Affiliations:** 1 State Key Laboratory of Rice Biology and Ministry of Agriculture Key Laboratory of Molecular Biology of Crop Pathogens and Insects, Institute of Biotechnology, Zhejiang University, Hangzhou 310058, China; yasmeen.abdallah@mu.edu.eg (Y.A.); 11716062@zju.edu.cn (M.L.); sollybombom@yahoo.com (S.O.O.); temoorahmed@zju.edu.cn (T.A.); 2 Department of Plant pathology, Minia University, Elminya 61519, Egypt; 3 Ministry of Agriculture Key Laboratory of Agricultural Entomology, Institute of Insect Sciences, Zhejiang University, Hangzhou 310027, China; dr_hatem@zju.edu.cn; 4 Department of Field Crop Pests, Plant Protection Research Institute, Agricultural Research Centre, Cairo 12619, Egypt; 5 Department of Dairy Microbiology, Animal Production Research Institute, Agriculture Research Centre, Dokki, Giza 12618, Egypt; amorbiotic@yahoo.com; 6 Institute of Plant Virology, Ningbo University, Ningbo 315211, China; yanchengqi@163.com; 7 State Key Laboratory for Managing Biotic and Chemical Treats to the Quality and Safety of Agro-products, Institute of Virology and Biotechnology, Zhejiang Academy of Agricultural Sciences, Hangzhou 310021, China; yangyong@zaas.ac.cn

**Keywords:** antibacterial activity, chitosan nanoparticles, green synthesis, ZnO nanoparticles, tomato extract

## Abstract

Bacterial leaf blight caused by *Xanthomonas oryzae* pv. *oryzae* (Xoo) is one of the most devastating diseases, resulting in significant yield losses in rice. The extensive use of chemical antibacterial agents has led to an increase the environmental toxicity. Nanotechnology products are being developed as a promising alternative to control plant disease with low environmental impact. In the present study, we investigated the antibacterial activity of biosynthesized chitosan nanoparticles (CSNPs) and zinc oxide nanoparticles (ZnONPs) against rice pathogen Xoo. The formation of CSNPs and ZnONPs in the reaction mixture was confirmed by using UV-vis spectroscopy at 300–550 nm. Moreover, CSNPs and ZnONPs with strong antibacterial activity against *Xoo* were further characterized by scanning and transmission electron microscopy, Fourier-transform infrared spectroscopy, and X-ray diffraction. Compared with the corresponding chitosan and ZnO alone, CSNPs and ZnONPs showed greater inhibition in the growth of Xoo, which may be mainly attributed to the reduction in biofilm formation and swimming, cell membrane damage, reactive oxygen species production, and apoptosis of bacterial cells. Overall, this study revealed that the two biosynthesized nanoparticles, particularly CSNPs, are a promising alternative to control rice bacterial disease.

## 1. Introduction

*Xanthomonas oryzae* pv. *oryzae* (Xoo) causes bacterial leaf blight in rice, which leads to severe yield losses and consequently alters the food supplies [1]. The restriction of the use of chemical pesticides has become a pressing necessity due to its environmental toxicity. In general, bacterial diseases of plants are very difficult to control and cause significant annual losses on a global scale. The use of agricultural antibiotics such as streptomycin and oxytetracycline have been restricted in most counties to avoid the development of antibiotic resistance, but have been recently enhanced in others. Interestingly, nanotechnology products are promising and environmentally friendly strategies for the control of bacterial diseases. In particular, significant attention has been paid to the development of a plant or microbe-based synthesis of metal nanoparticles [2,3,4,5] due to toxic compounds that have been found on the surface of the nanoparticles through the chemical synthesis method [6,7]. 

Furthermore, a high number of polyphenols, especially flavonoids [8] in tomato make it a good candidate for synthesizing nanoparticles. In addition, unlike red tomatoes, green tomato is rich in alkaloids and contain a high amount of ascorbic acid [9,10], the former was used as a biogenic stabilizer during the synthesis of chitosan nanoparticles (CSNPs) [11], while the latter plays a vital role in the production of zinc oxide nanoparticles (ZnONPs) [12]. Recently, numerous studies have demonstrated that the natural biopolymer chitosan and its derivative, as well as metal nanoparticles such as Ag, ZnO, TiO_2_, MgO, and CuO, have strong antibacterial activity against a variety of bacterial pathogen [13,14]. For example, Li et al. [15] reported that chitosan solution had strong antibacterial activity against *Xanthomonas* pathogenic bacteria isolated from *Euphorbia pulcherrima*, while the growth of the Xoo pathogen was significantly inhibited in our previous studies by the biosynthesized MgO nanoparticles [16], Ag nanoparticles [17,18] and ZnO nanoparticles [19]. Maruyama et al. [20] developed a system based on chitosan nanoparticles as carriers for the herbicides. Similarly, Campos et al. [21] prepared chitosan nanoparticles functionalized with β-cyclodextrin as a carrier system for botanical pesticides with a slower release profile.

The antimicrobial activity of the chitosan polymers and nanoparticles may be mainly attributed to their electric charge and high ability of adsorption as well as chemical reactions that allow them to interact efficiently with the bacterial cell membrane [22], while the inhibitory effect on the bacterial growth depends on their sizes and shapes as well as biological and structural properties [15]. Compared to the antibacterial activity of chitosan and metal nanoparticles alone, significant inhibition could be expected by the combination of chitosan with metal nanoparticles. Indeed, chitosan has also been studied as the main structural unit of nanomaterials due to its low toxicity, non-immunogenicity, and biodegradability. For example, [23] has synthesized and characterized chitosan/TiO_2_ nanocomposite with antibacterial activity against Xoo. 

However, the concerns are rising on the residue of heavy metal ion in the natural environment, which makes it necessary to exclude the heavy metal ion during the control of Xoo by the combination of chitosan with nanoparticles. Interestingly, chitosan nanoparticles (CSNPs) not only have less toxic impact compared to the chitosan and metal nanoparticles alone [24,25,26], but also shows an inhibitory effect on the growth of several microorganisms [27,28]. Indeed, much attention has been directed toward the synthesis of CSNPs, which have been applied in many fields such as nanomedicine and biomedical engineering [29] due to its small size, high permeability, biocompatibility, biodegradability, and cost-effectiveness [30,31,32]. Thus, it can be expected that the Xoo can be effectively inhibited by the synthesis of CSNPs.

The purpose of this research was to biosynthesize CSNPs with strong antibacterial activity against the rice pathogen Xoo, while ZnO nanoparticles (ZnONPs) were also biosynthesized as the positive control. Furthermore, the antibacterial activity of CSNPs was compared with that of chitosan, ZnO and ZnO nanoparticles alone. In addition, this study revealed the antibacterial mechanism of the biosynthesized CSNPs and ZnONPs against Xoo.

## 2. Materials and Methods 

### 2.1. Preparation of Aqueous Green Tomato Extract

Two grams of dried green tomatoes were placed in a 250 mL flask containing 200 mL deionized water and then boiled in a 60 °C water bath for 4 h. After filtering twice with filter paper Whatman no. 1, the aqueous green tomato extract was directly used in the installation of CSNPs and ZnONPs.

### 2.2. Green Synthesis of CSNPs and ZnONPs

CSNPs and ZnONPs were synthesized according to the method of Dipali et al. [11] with minor modifications. In brief, the chitosan-TPP mixture was obtained by dissolving 0.8 g of sodium tripolyphosphate (TPP) (Aladdin Industrial Co., Shanghai, China) in 100 mL of chitosan solution, (Sinopharm chemical Regent Co., Ltd., China) which were prepared by adding about 1.5 g of chitosan (C_6_H_11_NO_4_)_n_ from shrimp shells, ≥75% (deacetylated) in 200 mL of 2% acetic acid solution and then stirring under continuous magnetic for 30 min. Then, CSNPs were developed by adding 100 mL of the aqueous green tomato extract to 200 mL of a chitosan-TPP mixture dropwise under constant stirring for 30 min. Similarly, ZnONPs were developed by adding 100 mL of the aqueous green tomato extract to 100 mL of 1 M ZnO (Sangon Biotech Co., Ltd., Shanghai, China) and then stirring at 60 °C for 4 h. After discarding the supernatant through centrifugation at 10,000 g for 20 min, the pellets were washed with distilled water and then freeze-dried in ALPHA 1-2/LD-Plus vacuum to obtain the nanoparticles powders.

### 2.3. Characterization of CSNPs and ZnONPs with Strong Antibacterial Activities

#### 2.3.1. Analysis of UV-Vis Spectroscopy

The highest peak absorption levels of CSNPs and ZnONPs were measured according to [33] by using UV-Vis spectroscopy at 300–550 nm (Shimadzu spectrometer, Kyoto, Japan) with a length of 1 cm path. 

#### 2.3.2. TEM and SEM-EDS Observation

The morphology of two types of the nanoparticles CSNPs and ZnONPs with strong antibacterial activity was observed using transmission electron microscopy (TEM) (JEM-1230, JEOL, Akishima, Japan), which was carried out as described by Ahmed et al. [34] by fixing the film of the sample in a grid box. Furthermore, the morphology of two types of nanoparticles was also observed according to the method of Ibrahim et al. [17] using scanned electron microscopy (SEM) (TM-1000, Hitachi, Tokyo, Japan). In brief, a film was prepared on a grid of carbon-coated copper by fixing a little amount of sample on the grid. The film on the SEM grid was dried by placing the grid under the mercury lamp for 5 min. The device was connected with an energy dispersion spectrum (EDS) to confirm the presence of nanoparticles. 

#### 2.3.3. FTIR Analysis

The functional group of two types of the nanoparticles CSNPs and ZnONPs with strong antibacterial activity was determined based on the analysis of Fourier transform infrared spectroscopy (FTIR), which was carried out as described by Hossain et al. [35] by measuring the dried powder of the bio-synthesized two nanoparticles using Fourier transform infrared spectrometer (Vector 22, Bruker, Bremen, Germany) at a range of 500–4000 cm^−1^ regions at a resolution of 4 cm^−1^.

#### 2.3.4. XRD Analysis

The quality of two types of the nanoparticles CSNPs and ZnONPs with strong antibacterial activity was determined using Siemens X-ray diffractometer (XRD), which was performed as described by Abdallah et al. [16] using drop coated film of dried powder of two types of the nanoparticles on glass slides with operating conditions of 45 kV and 20 mA current with Cu-Ka radiation as an X-ray source in the 20–80° range at the 2θ angle.

### 2.4. Antibacterial Activity and Mechanism of Action

#### 2.4.1. Measurement of Bacterial Growth

The antibacterial activity of CSNPs and ZnONPs against Xoo strain GZ 0005 was measured on a solid medium by using agar well diffusion assay, which was carried out as described by Perez et al. [36] with minor modifications. In brief, 200 µL of overnight bacterial culture (approximately 1 × 10^8^ CFU/mL) was mixed with five mL of nutrient agar (NA) medium. About 50 µL of dried nanoparticle solutions at different concentrations (4.0, 8.0, and 16.0 μg/mL) were poured at the 6 mm wells and incubated for 24 h at 30 °C. Then the inhibition zone around the well was measured. The antibacterial activity of CSNPs and ZnONPs against Xoo strain GZ 0005 was also examined on liquid broth, which was performed as described by [37]. The sterile tubes were incubated at 30 °C with shaking (180 rpm) containing 100 µL of Xoo (approximately 1 × 10^8^ CFU/mL) in total 5 mL nutrient broth (Oxoid Ltd., Basingstoke, Hants, UK) to give different final concentrations of the nanoparticles (4.0, 8.0, and 16.0 µg/mL). The inhibitory effect on bacterial growth was determined by measuring the optical density at OD_600_ nm using a UV spectrophotometer after 48 h of incubation. The experiment was repeated twice, and each treatment had three replicates.

#### 2.4.2. Assay of Biofilm Formation

Biofilm formation of Xoo strain GZ 0005 was determined in a 96-well plate by measuring the absorption value at OD_570_ nm, according to Zhang et al. [38] with minor modifications. Briefly, each well was inoculated with 2 μL of overnight bacterial suspension and 198 μL NB broth supplement with CSNPs and ZnONPs with a final concentration of 0.0, 4.0, 8.0, and 16.0 μg/mL. The solutions were then incubated at 30 °C for 48 h without shaking to form a biofilm. After removing the supernatant, the wells were added with crystal violet for staining, while acetic acid (33%) was used to absorb crystal violet from the biofilm. Each treatment had at least six replicates and the experiment was repeated twice.

#### 2.4.3. Assay of Bacterial Motility

Swimming motility on plates was detected according to the method [39]. In brief, ten microliters of an overnight culture of Xoo strain GZ 0005 were spotted onto 0.8% (*w/v*) NA media, which was pre-mixed with CSNPs and ZnONPs with a final concentration of 0.0, 4.0, 8.0, and 16.0 μg/mL. After three days of incubation at 30 °C, bacterial migration areas were measured. Each treatment was expressed in triplicates and the experiment was repeated twice.

#### 2.4.4. TEM Observation of Bacterial Cells

Bacterial samples for TEM were prepared as described by Sun et al. [40] with some modifications. In brief, one mL of overnight bacterial culture of Xoo strain GZ 0005 was centrifuged at 11,000 rpm for 10 min, and the obtained pellet was suspended in CSNPs and ZnONPs solutions with the final concentration of 16 μg/mL, then incubated at 30 °C for 4 h. The samples were prepared following standard procedures for fixation and embedding then examined using an electron microscope H-7000FA (Hitachi, Tokyo, Japan) with an operating voltage of 75 kV.

#### 2.4.5. Flow Cytometric Analysis of Bacterial Cells

CSNPs and ZnONPs induced apoptosis was observed by flow cytometry (GALLIOS BECKMAN COULTER, Krefeld, Germany). After centrifuging the overnight culture of Xoo strain GZ 0005 (1 × 10^8^ CFU/mL) for 5 min at 5000 g, the bacterial pellets were treated with CSNPs and ZnONPs with the final concentration of 16.0 µg/mL for 4 h, then propidium iodide (PI) was added as described by Cai et al. [41] to stain bacterial nuclei. 

#### 2.4.6. Determination of Bacterial Reactive Oxygen Species (ROS)

Intracellular ROS was determined using fluorescent probe 2, 7-dichlorofluorescein diacetate (DCFH-DA), which interact with ROS to form the fluorescence trapped inside the cell. In brief, one mL overnight culture of Xoo strain GZ 0005 (1 × 10^8^ CFU/mL) was centrifuged for 5 min at 5000 g. Then, bacterial pellets were treated with CSNPs and ZnONPs with the final concentration of 16.0 μg/mL for 4 h, respectively, while rotenone was also applied before two types of nanoparticles. The negative control was treated with sterile water. After washing with sterilized water three times, 10 μM DCFH-DA was added and then incubated in the dark at room temperature for 30 min. Eventually, the samples were washed with sterilized water, and fluorescence was detected as described by Applerot et al. [42] using scanning confocal laser microscopy (SCLM) (Leica SP8, Germany). 

### 2.5. Statistical Analysis

Data were subjected to analysis of variance (ANOVA) using SAS 2003 software (SAS Institute, Cary, NC, USA). A general linear model (GLM) procedure was used to check the significant differences among the main treatments. Individual comparisons between mean values were performed using Duncan’s Method (*p* < 0.05).

## 3. Results and Discussion

### 3.1. Biosynthesis of CSNPs and ZnONPs

**Figure 1:**
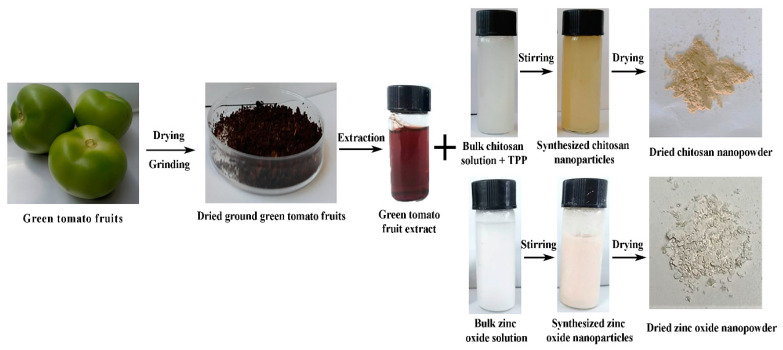
Schematic presentation of the preparation of chitosan nanoparticles (CSNPs) and zinc oxide nanoparticles (ZnONPs).

**Figure 2:**
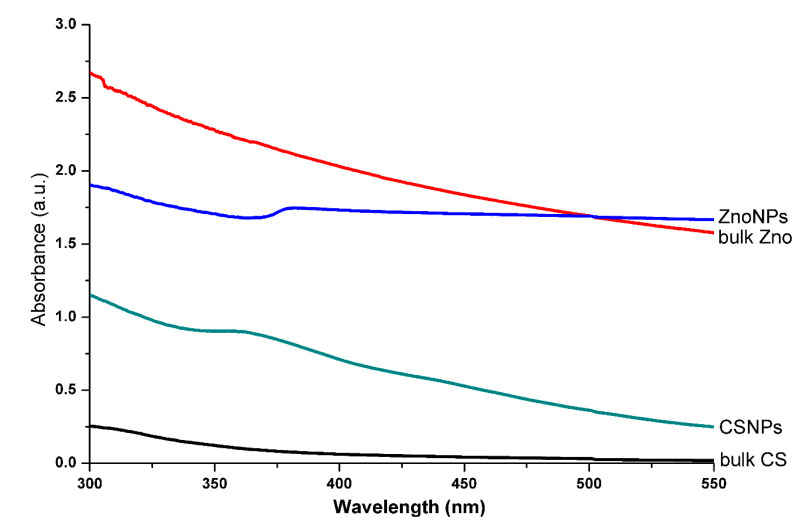
The UV-visible spectrum of bulk chitosan (CS), CSNPs, bulk ZnO, and ZnONPs solutions.

The biosynthesis procedure of two types of the nanoparticles by using dried green tomato extract as shown in the (Figure 1). Although peaks were not reached in both chitosan and ZnO solutions, there was a UV-Vis absorption spectra peak of 363 and 383 nm, corresponding to CSNPs and ZnONPs, respectively (Figure 2). In contrast, OH et al. [43] reported that the absorption peak wavelength was at 320 nm in the UV region for CSNPs, while Akhtar et al. [44] reported that the UV-Vis absorption spectra peak of ZnONPs was about 400 nm. However, in agreement with our study, Anumansirikul et al. [45] reported that the maximum peak for CSNPs was about 320–360 nm, whereas Ogunyemi et al. [19] found that the UV-vis spectra of zinc oxide nanoparticles showed a strong absorption band at 384, 380 and 386 nm for chamomile flower, olive leave, and red tomato fruit, respectively. As we know, the UV-Vis absorption spectrometry is an important technique for confirming the presence of nanoparticles in aquatic solution. Therefore, the inconsistent results in the UV-Vis absorption spectra peak may be mainly attributed to the difference in the procedure of biosynthesis of nanomaterials.

### 3.2. Characterization of CSNPs and ZnONPs 

In order to effectively apply the two biosynthesized nanoparticles in agriculture, we further characterized the CSNPs and ZnONPs with strong antibacterial activity by TEM and SEM-EDS observation as well as FTIR and XRD analysis.

#### 3.2.1. TEM and SEM-EDS Observation

**Figure 3:**
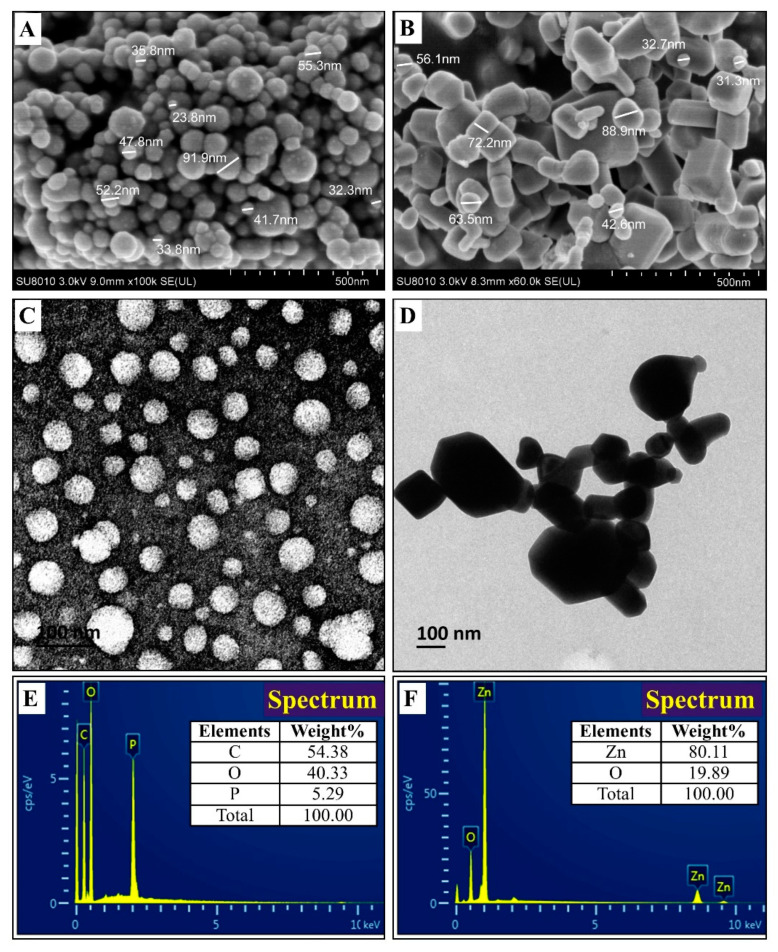
Characterization of the bio-synthesized by SEM analysis: (**A**) CSNPs; (**B**) ZnONPs, TEM analysis; (**C**) CSNPs; (**D**) ZnONPs and EDS; (**E**) CSNPs; (**F**) ZnONPs.

The size and shape of nanoparticles play an important role in their antimicrobial activity against microbial pathogens, while smaller particle size can help nanoparticles easily enter the cell wall of microorganisms and increase the uptake of vehicles into the microbial cell [46]. Similar to the result of Parida et al. [47], the result of TEM and SEM images indicated that the biosynthesized CSNPs have a spherical shape with particle size ranging from 23.8 to 91.9 nm (Figure 3A,C). These results are in agreement with the data provided by Ilk et al. [48], who obtained similar results of CSNPs with spherical in shape and size ranging from 200 to 350 nm in diameter. In the case of ZnONPs, TEM and SEM analysis showed diverse polymorphic shapes such as spherical and hexagonal shapes with particle size ranging from 31.3 to 88.9 nm (Figure 3B,D). Similar results have been reported by Fu et al. [49], who synthesized hexagonal rod shape ZnONPs by using *Plectranthus amboinicus* leaf extract.

The elemental composition of the biosynthesized CSNPs and ZnONPs was confirmed by using EDS analysis. The EDS spectra revealed the percentage elemental composition of CSNPs, composed of carbon (54.38%), oxygen (40.33%), and phosphorus (5.29%) (Figure 3E). In the case of ZnONPs, EDS peaks showed the percentage elemental composition, consisting of zinc (80.11%) and oxygen (19.89%) (Figure 3F). Furthermore, the elemental percentage of CSNPs in this study is similar to the report of Sotelo et al. [50], while the elemental percentage of ZnONPs of this study is similar to the report of Ogunyemi et al. [19]. In agreement with the result of Ali et al. [51], the EDS analysis obtained in this study shows that the samples prepared using aqueous green tomato extract contained pure ZnONPs.

#### 3.2.2. FTIR Analysis

**Figure 4:**
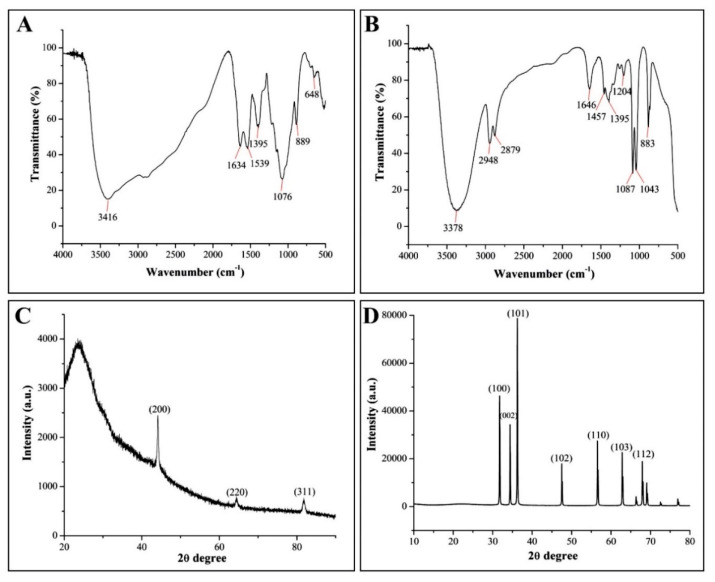
Fourier-transform infrared spectrum of (**A**) CSNPs and (**B**) ZnONPs. X-Rays diffraction patterns of (**C**) CSNPs and (**D**) ZnONPs.

The stability of green nanoparticles may be, at least partially, due to the capping proteins, which could have an additional advantage as antimicrobial agents. Indeed, the results of the FTIR spectrum provide an interpretation of the correlation between the absorption bands and the chemical compounds [52], which makes it possible for us to understand which biomolecules are involved in the increased antibacterial activity of two types of the nanoparticles. In the current study, the FTIR spectra of CSNPs showed multiple absorption peaks at 3416, 1634, 1539, 1395, 1076, and 889 cm^−1^ (Figure 4A). The absorption peaks at 3416 and 1634 cm^−1^ represented the O-H stretching group of alcohol and C=C stretching group of conjugated alkene, respectively. The absorption peaks observed at 1539 and 1395 cm^−1^ were due to the N-O stretching group of a nitro compound and O-H bending group of carboxylic acid, respectively. Furthermore, the peaks detected at 1076 and 889 cm^−1^ were attributed to the C-O stretching group of primary alcohol and the C=C bending group of alkene, respectively. In agreement with the data of this study, Salehizadeh et al. [53] reported that the FTIR spectra of CSNPs commonly show a peak at around 3416 cm^−1^ that corresponds to O-H and –NH stretching vibration in chitosan. 

In the case of ZnONPs, FTIR analysis indicated several absorption peaks at 3378, 2948, 2879, 1646, 1457, 1395, 1204, 1078, and 1043 cm^−1^, together with a weak peak at 889 cm^−1^, showing the complexity in capping pattern (Figure 4B). The FTIR peaks of ZnONPs at 3378 cm^−1^ were due to the N−H stretching group of aliphatic primary amine. The peaks at 2948 and 2879 cm^−1^ represented the presence N-H stretching group of amine salt, while absorption peaks at 1646 and 1457 cm^−1^ were attributed to the C=N stretching group of imine/oxime and C=C aromatic, respectively. The peaks at 1395 and 1204 cm^−1^ revealed the O-H bending group of carboxylic acid and C-O stretching group of alkyl aryl ether, respectively, whereas, the absorption peaks at 1078 and 1043 cm^−1^ were attributed to C-O stretching group primary alcohol and S=O stretching group of sulfoxide, respectively. In agreement with the result of this study, previous studies reported the similar FTIR peaks for ZnONPs [54,55].

#### 3.2.3. XRD Analysis

As shown in Figure 4, the crystalline nature and crystallite planes of two types of nanoparticles were justified based on the XRD spectrum peaks. Indeed, the typical distinct crystalline peaks were observed at 2θ = 44.31°, 64.45°, and 81.77°, which were assigned to (200), (220), and (311) reflections for green CSNPs (Figure 4C). On the contrary, Yen et al. [56] reported the peak of chitosan at 2θ = 20.7°. In some cases, due to the transformation of structure into an amorphous nature, the peak disappears at 2θ and the emergence of broad bands at 2θ = 35° [57] XRD patterns of ZnONPs showed peaks at 2θ = 31.74°, 34.40°, 36.22°, 47.51°, 56.65°, 62.83°, and 69.05°, which were assigned to (100, 002, 101, 102, 110, 103, and 112) (Figure 4D). In the previous study, Ogunyemi et al. [19] reported the similar XRD diffraction peaks at 2θ = 31.8°, 34.5°, 36.3°, 47.6°, 56.6°, 62.9°, 68.0°, 72.6°, 77.0°, 81.4°, and 89.7° of ZnONPs synthesized by using *Matricaria chamomilla* plant extracts. The diffraction peaks have been keenly indicated to the hexagonal structure of ZnONPs. The narrow width of ZnO diffraction peaks in the XRD pattern proves that the samples were well crystallized [58]. Furthermore, the Sherrer’s equation (particle size = kλ/b cos θ) revealed that the average particle size of CSNPs and ZnONP were 27.8 and 60.3 nm, respectively. The current results are in agreement with previous studies, who observed the similar average size of CSNPs and ZnONPs through XRD analysis [31,59].

### 3.3. Antibacterial Activity of CSNPs and ZnONPs

#### 3.3.1. In Vitro Inhibitory Effect on the Growth of Xoo

**Figure 5:**
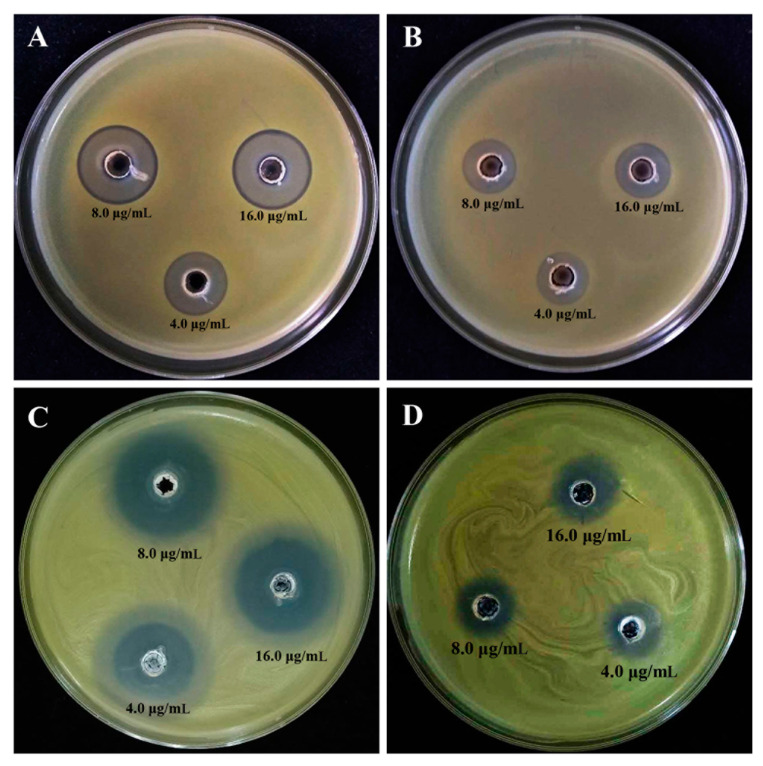
The in vitro inhibitory effect of (**A**) CSNPs, (**B**) bulk CS, (**C**) ZnONPs, and (**D**) bulk ZnO on the growth of *Xanthomonas oryzae* pv. *oryzae* strain GZ0005 following 24 h of incubation at 30 °C.

The results from this study indicated that the growth of Xoo strain GZ 0005 on the NA medium was effectively inhibited by bulk chitosan, bulk ZnO, CSNPs, and ZnONPs, while a greater effect was observed in the latter two nanoparticles (Figure 5 and Table 1). Indeed, CSNPs at the final concentration of 4.0, 8.0, and 16 μg/mL showed a mean inhibitory area of 2.1, 2.3, and 2.4 cm compared with that of 0.9, 1.2, and 1.3 cm from the normal chitosan, respectively. Furthermore, the dried ZnONPs powder at the final concentration of 4.0, 8.0, and 16 μg/mL showed an inhibitory area of 2.4, 2.6, and 2.9 cm, compared with that of 1.4, 1.5, and 1.8 cm from bulk ZnO, respectively. The inhibitory effect of CSNPs on the growth of Xoo in NB broth was determined by calculating the rate of inhibition in optical density (OD) at 600. Indeed, compared to the negative control, CSNPs at the final concentration of 4.0, 8.0, and 16.0 μg/mL showed an inhibition rate of 86.28, 86.76, and 86.85%, while ZnONPs at the final concentration of 4.0, 8.0, and 16.0 μg/mL showed an inhibition ratio of 59.39, 73.93, and 86.66%, respectively (Figure 5 and Table 2).

In agreement with several previous reports [17,23], the greater antibacterial activity could be achieved by the combination of nanoparticles with bulk chitosan and a heavy metal salt. Furthermore, there was no significant difference in the inhibitory zone on the NA medium between two types of nanoparticles, CSNPs and ZnONPs. However, CSNPs showed a greater inhibition on the growth of Xoo in NB broth compared to that of ZnONPs, indicating that Xoo strain GZ 0005 was more sensitive to CSNPs than to ZnONPs. As described in our previous studies, the biosynthesized silver nanoparticles showed promising antibacterial activity against rice pathogen Xoo [16,17]. On the other hand, CSNPs seem to be also safer than metallic NPs e.g., silver, copper, Zinc, etc. [19,60,61], due to the absence of the heavy metal ion, which poses a potential risk to plant growth and microbial activity in soils. In agreement with the result of this study, Li et al. [22] reported the antibacterial activity of chitosan/TiO_2_ nanocomposite against Xoo. However, it can be inferred from this study that two types of nanoparticles, in particular, CSNPs, have a good prospect to be used as antibacterial agents again rice bacterial pathogen.

#### 3.3.2. Inhibition of Biofilm Formation and Motility Swimming

**Figure 6:**
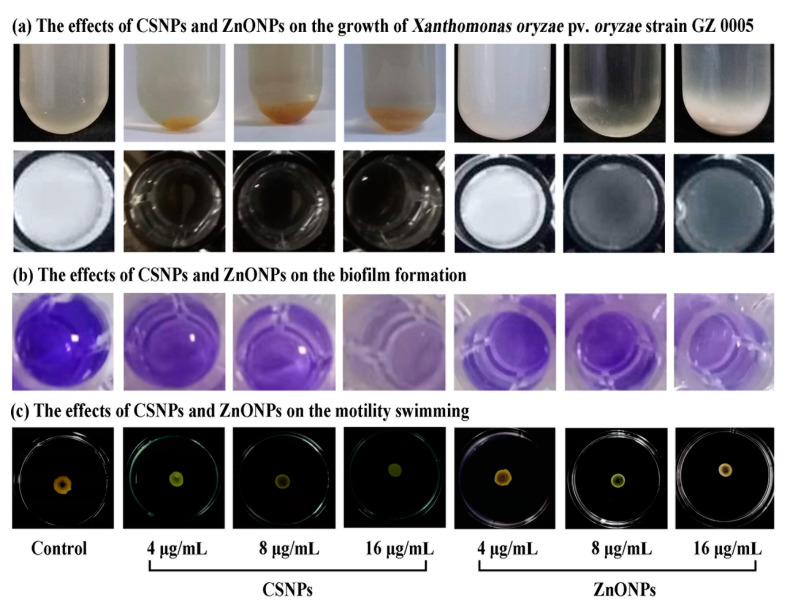
The inhibitory effects of CSNPs and ZnONPs at different concentrations (4, 8, and 16 μg/mL) on (**a**) growth, (**b**) biofilm formation, and (**c**) motility swimming of *Xanthomonas oryzae* pv. *oryzae* strain GZ 0005.

The results from this study indicated that the OD_570_ value of Xoo strain GZ 0005 was 1.13 after 48 h of incubation, while the diameter of migration areas was 18.7 mm after 3 days of incubation in the absence of the two biosynthesized nanoparticles. However, biofilm formation and motility swimming of Xoo strain GZ 0005 were significantly reduced by the two biosynthesized nanoparticles. Indeed, CSNPs at the concentration of 4.0, 8.0, and 16.0 μg/mL caused a 50.44%, 61.91%, and 69.91%, respectively, reduction in the OD_570_ value, and a 10.7%, 19.8%, and 32.1%, respectively, reduction in the diameter of migration areas of Xoo strain GZ 0005 (Figure 6 and Table 2). Similarly, ZnONPs at the concentration of 4.0, 8.0, and 16.0 μg/mL caused a 62.83%, 69.91%, and 76.10%, respectively, reduction in the OD_570_ value, and a 5.3%, 14.4%, and 25.1%, respectively, inhibition in the diameter of migration areas of Xoo strain GZ 0005 compared with the control after 48 h of incubation (Figure 6 and Table 2).

In general, this result indicated that the inhibitory effect in biofilm formation and motility swimming correlated positively with the concentration of two types of nanoparticles. The biofilms and motility swimming was able to protect the bacteria from immune responses by the host and contribute to bacterial resistance during host–pathogen interaction [62]. In agreement with the result of this study, de Paz et al. [63] reported the antimicrobial effect of chitosan nanoparticles on *Streptococcus mutans* biofilms, Tamara et al. [64] proposed that chitosan nanoparticles might have an impact on the pathways involved in the regulation of motility and biofilm formation, Lee et al. [65] indicated that ZnO nanoparticles could inhibit biofilm formation and virulence factor production of *Pseudomonas aeruginosa*. Therefore, the antibacterial activity of two types of nanoparticles against Xoo strain GZ 0005 may, at least partially, attribute to their inhibition in biofilm formation ability and motility swimming.

#### 3.3.3. TEM Observation of Cell Damage

**Figure 7:**
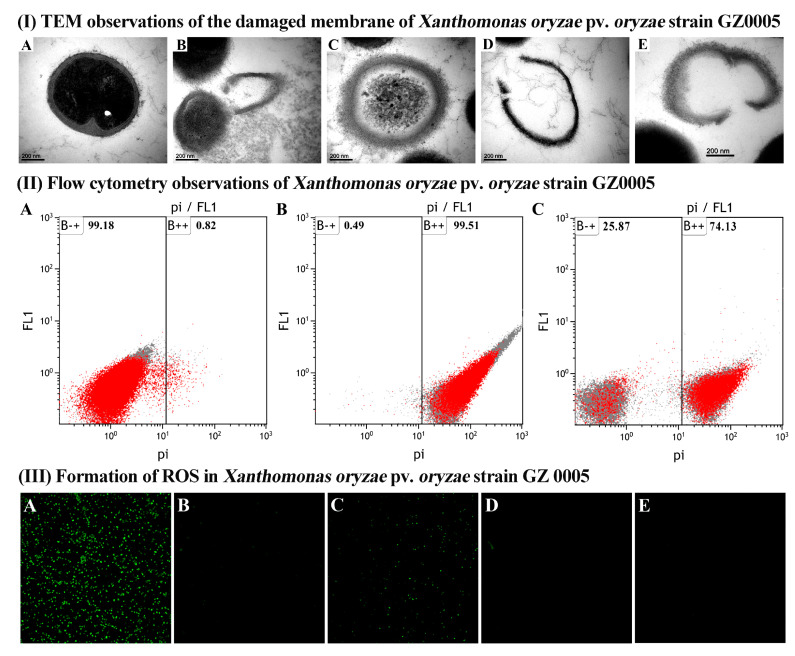
(**I**) TEM observations of the damaged membrane of *Xanthomonas oryzae* pv. *oryzae* strain GZ0005 treated with (A) sterile water, (B,C) CSNPs at 16 μg/mL, and (D,E) ZnONPs at 16 μg/mL. (**II**) Flow cytometry observations of *Xanthomonas oryzae* pv. *oryzae* strain GZ0005 cells after incubation with (A) sterile water, (B) CSNPs at 16 μg/mL, and (C) ZnONPs at 16 μg/mL. (**III**) Formation of ROS in *Xanthomonas oryzae* pv. *oryzae* strain GZ 0005 cells. (A) 4 h of incubation with CSNPs, (B) rotenone was added before 4 h of incubation with CSNPs, (C) 4 h of incubation with ZnONPs, (D) rotenone was added before 4 h of incubation with ZnONPs, and (E) 4 h of incubation with sterile water.

TEM observation of Xoo strain GZ 0005 showed an intact and clear cell wall with an evenly dispersed cell content that corresponds to the proteins, DNA and cytoplasmic content of the bacterial cells in the absence of the two biosynthesized nanoparticles CSNPs and ZnONPs. In contrast, microscopic images of Xoo cells treated with the biosynthesized CSNPs and ZnONPs at the final concentration of 16.0 μg/mL showed extensive destruction of cell walls compared to the negative control treated with distilled water. Indeed, the synthesized nanoparticles not only make the cell wall and cytoplasmic membrane became wrinkled, broken, and abnormal, but also caused the leakage of nutrients and nucleic materials, resulting in bacterial death (Figure 7).

In agreement with the result of this study, the damage of chitosan and zinc oxide nanoparticle to bacterial cells has been well studied [66,67,68]. In addition, many studies have reported that the primary mechanism for the inhibitory effect may be mainly due to the interaction between the positive charges of nanoparticles and the negatively charged compounds in the bacterial cell wall, which caused the penetration of nanoparticles into the bacterial cells, increasing membrane permeability, and intracellular flow [69,70,71,72]. Therefore, it could be suggested that the antibacterial activity of two nanoparticles against Xoo strain GZ 0005 may be, at least partially, attributed to the destruction of the cell wall, which is an essential structure for the life of bacteria.

#### 3.3.4. FC Observation of the Apoptosis of Xoo Cells

In addition to microscopic images of bacterial cells, a rapid and accurate assessment of cell damage was also observed in FC analysis using the propidium iodide (PI), which is a type of fluorochrome that can pass through damaged or dead cells, while the DNA stain was detected using ultraviolet rays. In agreement with the result of TEM observation, result from this study indicated that the two biosynthesized nanoparticles CSNPs and ZnONPs markedly increased the apoptosis of Xoo cells to 99.51% and 74.13%, respectively, compared to 0.82% for the negative control treated with distilled water (Figure 7). Collectively, the data presented cell injury or death when bacteria were treated with the CSNPs and ZnONPs. 

In agreement with the result of this study, cell membrane damage and promoted flowage was observed in *Escheria coli* following the treatment with nanoparticles and detection by FC analysis [73]. Additionally, Parida et al. [47] reported high apoptosis in cancer cells, which were exposed to different concentrations of chitosan nanoparticles. Furthermore, this result indicated that CSNPs has a greater effect on cell damage than that of ZnONPs, which is consistent with their inhibitory effect on the growth of Xoo strain GZ 0005 when incubating the bacteria in NB broth. Thus, it can be inferred that the antibacterial activity of the two biosynthesized nanoparticles CSNPs and ZnONPs against Xoo strain GZ 0005 may, at least partially, due to apoptosis of bacterial cells.

#### 3.3.5. CLSM Observation of ROS Generation

Generation of reactive oxygen species (ROS) in Xoo strain GZ 0005 cells was determined based on CLSM observation of the intensity of the fluorescence after 4 h of incubation. Results from this study indicated that no obvious fluorescence was observed in bacterial cells in the negative control treated with sterile water. Furthermore, a little fluorescence was observed in Xoo strain GZ 0005 cells treated with 16.0 μg/mL of ZnONPs, while a lot of fluorescence was observed in Xoo strain GZ 0005 cells treated with 16.0 μg/mL of CSNPs (Figure 7). However, all fluorescence disappeared when the ROS inhibitor rotenone was applied in Xoo strain GZ 0005 cells before treatment with 16.0 μg/mL of the two biosynthesized nanoparticles CSNPs and ZnONPs (Figure 7). 

In agreement with the result of this study, Banerjee et al. [74] found that chitosan-Ag nanocomposite was able to generate ROS in *E. coli* cells. The formation of ROS promotes the oxidative stress in the cells, which may in turn induce cell damage, causing cell lysis or distortion of bacterial membranes, resulting in the leakage of DNA and protein, even bacterial death [75]. Indeed, some studies have indicated that the antibacterial activity of ZnONPs could be due to the generation of ROS on oxides surfaces, which induced significant morphological changes and outflow in bacterial cells [66,76]. On the other hand, rotenone has been regarded as a scavenger of the electron transport chains in mitochondria, which is an essential site in the generation of ROS [77], this explains why a significant decrease in ROS content by the rotenone. Our results indicated that the strong antibacterial activity of the two biosynthesized nanoparticles, in particular, CSNPs was, at least partially, due to the generation of ROS.

## 4. Conclusions

In conclusion, this study successfully biosynthesized CSNPs and ZnONPs, which showed a stronger inhibitory effect on the growth of rice pathogenic bacteria Xoo compared to that of bulk chitosan or bulk ZnO, respectively. Furthermore, the two synthesized nanoparticles with strong antibacterial activity were characterized by the analysis of UV–Vis spectroscopy, XRD, and FTIR as well as TEM and SEM observation. In addition, the antibacterial activity of two types of the nanoparticles may be able to, at least partially, be attributed to apoptosis, generation of ROS, reduction in biofilm formation and swimming, destruction or disintegration of the cell walls, and leakage of the intracellular contents, which eventually resulted in cell death. Overall, it could be concluded from this study that the two synthesized nanoparticles particularly CSNPs have a great potential to suppress Xoo infections in rice.

## Figures and Tables

**Table 1:** The inhibitory effect of bulk chitosan (CS), chitosan nanoparticles (CSNPs), bulk ZnO, and zinc oxide nanoparticles (ZnONPs) on the growth of *Xanthomonas oryzae* pv. *oryzae* strain GZ0005 in nutrient agar plates.

Treatments	Inhibition Zone (cm)
Xoo+	
Bulk CS (4 µg/mL)	0.9 ± 0.15 ^b^
Bulk CS (8 µg/mL)	1.2 ± 0.26 ^b^
Bulk CS (16 µg/mL)	1.3 ± 0.42 ^b^
Xoo+	
CSNPs (4 µg/mL)	2.1 ± 0.42 ^a^
CSNPs (8 µg/mL)	2.3 ± 0.21 ^a^
CSNPs (16 µg/mL)	2.4 ± 0.26 ^a^
Xoo+	
Bulk ZnO (4 µg/mL)	1.4 ± 0.16 ^b^
Bulk ZnO (8 µg/mL)	1.5 ± 0.16 ^b^
Bulk ZnO (16 µg/mL)	1.8 ± 0.16 ^b^
Xoo+	
ZnONPs (4 µg/mL)	2.4 ± 0.21 ^a^
ZnONPs (8 µg/mL)	2.6 ± 0.21 ^a^
ZnONPs (16 µg/mL)	2.9 ± 0.10 ^a^

Bulk chitosan and chitosan nanoparticles were dissolved in acetic acid, while bulk ZnO and zinc oxide nanoparticles were dissolved in ethylene glycol. The inhibition zones are represented as the means ± standard error of at least three independent experiments. Different letters within one column indicate significant differences between treatments (*p* ≤ 0.05).

**Table 2:** The in vitro inhibitory effect of chitosan nanoparticles (CSNPs) and zinc oxide nanoparticles (ZnONPs) at different concentrations (4, 8, and 16 µg/mL) on the growth, biofilm formation, and motility of *Xanthomonas oryzae* pv. *oryzae* strain GZ0005.

Treatments	Bacterial Growth	Biofilm Formation	Motility (mm)
Xoo	1.65 ± 0.02 ^a^	1.13 ± 0.03 ^a^	18.7 ± 0.02 ^a^
Xoo + CSNPs (4 µg/mL)	0.14 ± 0.00 ^b^	0.56 ± 0.05 ^b^	16.7 ± 0.47 ^b^
Xoo + CSNPs (8 µg/mL)	0.14 ± 0.00 ^b^	0.43 ± 0.02 ^bc^	15.0 ± 0.82 ^c^
Xoo + CSNPs (16 µg/mL)	0.14 ± 0.00 ^b^	0.34 ± 0.02 ^c^	12.7 ± 0.94 ^d^
Xoo + ZnONPs (4 µg/mL)	0.67 ± 0.02 ^b^	0.42 ± 0.01 ^b^	16.2 ± 0.47 ^b^
Xoo + ZnONPs (8 µg/mL)	0.43 ± 0.02 ^c^	0.34 ± 0.00 ^bc^	17.3 ± 0.82 ^c^
Xoo + ZnONPs (16 µg/mL)	0.22 ± 0.04 ^d^	0.27 ± 0.00 ^d^	14.5 ± 0.82 ^d^

Chitosan nanoparticles were dissolved in acetic acid, while zinc oxide nanoparticles were dissolved in ethylene glycol. Bacterial growth and biofilm formation were determined by measuring the value of OD_600_ and OD_570_, respectively. Different letters within one column indicate significant differences between treatments (*p* ≤ 0.05).
